# Eating Disorders in Pregnancy

**DOI:** 10.1192/j.eurpsy.2022.77

**Published:** 2022-09-01

**Authors:** N. Micali

**Affiliations:** University of Geneva, Psychiatry, Geneva, Switzerland

**Keywords:** post-partum, Eating Disorders, identification, Pregnancy

## Abstract

Eating Disorders are common psychiatric disorders, and their occurrence is not rare in pregnancy. The aim of this presentation will be to provide an overview of eating disorders in pregnancy. I will first cover prevalence and nature of eating disorders and their symptoms in pregnancy. I will present quantitative and qualitative data from my own research. I will then present research on the effects of eating disorders on pregnancy and obstetric outcomes. I will conclude discussing issues around identification of eating disorders in pregnancy; in particular research from my lab on gaps in identification, and how to improve identification of eating disorders in pregnancy.

**Disclosure:**

No significant relationships.

